# Comparison of cellular responses of cultured fibroblasts from Iriomote wild cats and domestic cats exposure to polyinosinic:polycytidylic acid

**DOI:** 10.1371/journal.pone.0332954

**Published:** 2025-09-25

**Authors:** Masafumi Katayama, Ayusa Kataoka, Tomokazu Fukuda, Manabu Onuma

**Affiliations:** 1 Biodiversity Division, National Institute for Environmental Studies, Tsukuba, Ibaraki, Japan; 2 Ayutomo Animal Hospital, Kitakyusyu, Fukuoka, Japan; 3 Graduate School of Science and Engineering, Iwate University, Morioka, Iwate, Japan; UTRGV: The University of Texas Rio Grande Valley, UNITED STATES OF AMERICA

## Abstract

The Iriomote wild cat is endemic to Iriomote Island. The total population is only 100 cats. These cats are thus listed as critically endangered on the IUCN Red List. Infectious disease is a risk factor for further reducing the Iriomote wild cat population. *RIG-I*, *MDA5* and *TLR3* are important sensors that recognize host viral defense responses. *IL6*, *Mx*, and *OAS* are downstream genes of *RIG-I* and *MDA5.* Polyinosinic:polycytidylic acid (poly(I:C)) stimulates the *RIG-I*, *MDA5* and *TLR3* in many animals. In this study, we thus tried to compare *RIG-I*, *MDA5,* and *TLR3* responses after exposure to the poly(I:C) in domestic cat- and Iriomote wild-cat-derived fibroblasts. This study also compared the expression of downstream gene (*IL6*, *Mx*, and *OAS*) of *RIG-I* and *MDA5.* This study analyzed the genes expressions of domestic cat- and Iriomote wild-cat-derived fibroblasts after exposure the poly(I:C), using real-time PCR and RNA-seq. In addition to these genes expression, this study compared the gene sequence of *RIG-I* and *MDA5* between domestic cat and Iriomote wild cat with information of public database. *RIG-I* and *MDA5* levels were increased in both domestic cat- and Iriomote cat-derived fibroblasts after poly(I:C) exposure, while *TLR3* expression did not change dramatically after poly(I:C) exposure. A comprehensive gene expression analysis revealed that molecular responses of fibroblasts derived from domestic cats and those derived from Iriomote wild cats to poly(I:C) exposure were similar. Furthermore, *RIG-I* and *MDA5* sequences are similar between domestic and Iriomote wild cat. Our results suggested that poly(I:C) mainly stimulates *RIG-I* and *MDA5* among the three genes (*RIG-I, MDA5* and *TLR3*) examined in this study, and the *RIG-I* and *MDA5* mediated molecular responses to poly(I:C) exposure are similar in domestic and Iriomote wild cats fibroblasts. To the best of our knowledge, this is the first study to compare the *RIG-I* and *MDA5* responses of Iriomote wild cats and domestic cats after poly(I:C) exposure. This study provides valuable insights into the cellular response mediated by *RIG-I* and *MDA5* in the Iriomote wild cat.

## Introduction

UNESCO has registered Iriomote Island as a World Natural Heritage Site because many endemic species and/or subspecies (e.g., *Cistoclemmys flavomarginata evelynae, Neocaridina iriomotensis*) inhabit Iriomote Island (https://whc.unesco.org/ja/list/1574). The Iriomote wild cat (*Prionailurus bengalensis iriomotensis*) is a subspecies endemic to the Iriomote Islands [1]. The Iriomote wild cat is located at the top of the food chain on the Iriomote Islands. Therefore, a reduction in the number of Iriomote wild cats will exert critical effects on the ecosystem of the Iriomote Islands.

The Iriomote wild cat is listed as critically endangered on the IUCN Red List [2]. The number of Iriomote wild cats is approximately 100, thus a decrease in their population could directly lead to extinction [1]. Listed representative risk factors include traffic accidents, domestic cat-derived infectious diseases (such as Feline Immunodeficiency Virus), habitat destruction, and negative influences of introduced species [1]. Infectious diseases can spread rapidly, causing a high rate of deaths. Therefore, countermeasures against infectious diseases impacting the Iriomote wild cat are highly desirable for conservation of the Iriomote wild cat.

Many domestic cats die from infectious diseases. For example, approximately 8,000 domestic cats died from feline coronavirus (FCoV) infection in the Republic of Cyprus for during half a year [3]. In addition to FCoV, influenza is considered a high-risk factor for mass mortality in domestic cats. In Poland, abnormal domestic cat deaths have been attributed to Influenza A (H5N1) infections [4]. In Japan, severe fever with thrombocytopenia syndrome (SFTS) has been reported as a lethal infectious disease in domestic cat [5,6]. The Iriomote wild cat is genetically close to the domestic cat because it is a member of the *Felidae* family. These infectious diseases might lead to the reduction of individual numbers of Iriomote wild cat, as same as domestic cat.

Furthermore, veterinarians vaccinate a number of domestic cats due to the maintenance provided by their owners. In contrast to domestic cats, veterinarians have not vaccinated the Iriomote wild cat due to the wildlife individual. The vaccination is a powerful tool for the protection of the host from infectious diseases, including viruses. Therefore, the Iriomote wild cat would be more vulnerable to infectious diseases than domestic cats of companion animals.

Members of the retinoic acid-inducible gene I (RIG-I)-like receptor (RLR) family (*RIG-I* and *MDA*) are observed in the cytoplasm, and *RIG-I* and *MDA5* are important sensors for viral recognition [7–9]. As representative downstream genes of *RIG-I* and *MDA5, IL6*, *Mx*, and *OAS* are known. Several previous studies have suggested that *RIG-I* and *MDA5*-derived signals have a critical effect on the exacerbation of infectious diseases with the virus in animals. As a case in point, chickens lack the *RIG-I* gene, and this lack is regarded as a primary reason for their elevated sensitivity to the influenza virus [10–12]. In the flying fox, *RIG-I* and *MDA5* gene expression is significantly increased upon RNA virus infection. This observation provides clues regarding the mechanism by which the Flying Fox is a natural host for viruses [13]. Our group has reported that the *MDA5* gene is mutated and non-functional in the Okinawa rail, and that the innate immune response of the Okinawa rail is delayed compared to that of the chicken [14]. This result suggested that Okinawa rail might be weak with infectious disease. Therefore, we considered that the response of *RIG-I* and *MDA5* after the recognition of virus RNA might be the clue of the elucidation in the sensitivity of infectious disease in host animals.

To evaluate the response of *RIG-I*, *MDA5, TLR3 and downstream genes* of *RIG-I* and *MDA5* (e.g., *IL6*, *Mx*, and *OAS*) of Iriomote wild cat, in vivo experiment would be the first choice, while the barrier of in vivo experiment is very high due to the endangered animals. Then, we focused on cultured fibroblast. Fibroblasts can be obtained from dead individuals, obviating the necessity of sacrificing individuals. *RIG-I* and *MDA5* are expressed in mammalian and avian fibroblasts. Many studies have thus used fibroblasts to analyze *RIG-I* and *MDA5* mediated response genes [8,11,14,15]. These data suggest that cultured fibroblasts can be useful for analyzing *RIG-I* and *MDA5* mediated responses. We therefore hypothesized that poly:IC would stimulate domestic and Iriomote wild-cat-derived fibroblasts.

In this study, we characterized *RIG-I*, *MDA5, TLR3 and downstream genes* of *RIG-I* and *MDA5* (e.g., *IL6*, *Mx*, and *OAS*) mediated gene stimulation in response to poly(I:C) exposure using fibroblasts from domestic cats and Iriomote wild cats. Poly(I:C) is an analog of double-stranded RNA [16]. Similar to an RNA virus, poly(I:C) can stimulate *RIG-I* and *MDA5* in various animal’s cultured fibroblast [11,17–19]. We thus hypothesized that poly(I:C) will stimulate *RIG-I* and *MDA5* in domestic cat- and Iriomote wild cat-derived fibroblasts. After poly(I:C) exposure, we performed a comprehensive gene expression analysis of domestic cat- and Iriomote wild cat-derived fibroblasts.

## Materials and methods

### Domestic cat- and Iriomote wild cat-derived fibroblasts

Domestic cat skin samples were obtained from surgeries performed at veterinary clinics. Informed consent for use of domestic cat tissue samples for academic research was obtained from the domestic cats’ owners. This study used three domestic cat-derived skin fibroblasts. When the veterinarian performed the castration surgery, a small piece of skin was removed from the cats. Therefore, those skin samples were obtained from castration surgeries performed at veterinary clinics on healthy male cats. All domestic cats are categorized in Felidae (family) Felis (Genus). In contrast to the domestic cat, Iriomomte wild cats are categorized in Felidae (family), Prionailurus (Genus). Therefore, both the domestic cat and the Iriomote wild cat are categorized as the same family, but not the same genus. Therefore, individual differences among the species would be smaller than inter-genus differences. We also used ovary-derived fibroblasts (S1 Fig). These domestic cats contained the mixed-breed cat.

Iriomote wild cat-derived skin tissues were obtained from the Iriomote Wildlife Conservation Center (https://iwcc.jp/english/). The staff of the Iriomote Wildlife Conservation Center sometimes find dead Iriomote cats on Iriomote Island. On these occasions, they collect skin tissue samples and send them to the National Institute of Environmental Studies (NIES, Tsukuba, Japan). The details described below exclude the exact sampling locations to protect animals against poaching. All records are available from the National Institute for Environmental Science (NIES). In this study, we used three individual derived fibroblast cells.

A dead Iriomote wild cat was identified on March 28, 2016. This dead Iriomote wild cat was transported to the National Institute for Environmental Studies (NIES) on May 11, 2016, and cultured fibroblasts were obtained from its skin (NIES ID: 3786M). Another dead Iriomote wild cat was observed on May 4, 2016. This animal was transported to the National Institute for Environmental Studies (NIES) on May 11, 2016, and cultured fibroblasts were obtained from its skin (NIES ID: 3787M). A third dead Iriomote wild cat was observed on May 31, 2016. Its body was transported to the National Institute for Environmental Studies (NIES) on June 6, 2016, and cultured fibroblasts were obtained from its skin (NIES ID: 4355M).

For this study, we obtained our cells from surgical tissues (domestic cats) or dead animals (Iriomote cats). Therefore, we did not submit an animal experiment approval protocol based on the ethical standards of the National Institute of Environmental Studies (NIES, Tsukuba, Japan). The population sizes of Iriomote wild cats are estimated to be no more than 100 individuals [1]. Therefore, the opportunity of obtaining a somatic cell is quite rare. The genetic background would be close to all three individuals of this study, since the Iriomote cat showed a remarkable reduction in genetic diversity in mtDNA and microsatellite variation [1].

### Cell culture

Domestic cat- and Iriomote cat-derived tissues were placed on gelatin-coated dishes and cultured in Dulbecco’s modified Eagle’s medium (DMEM + GlutaMax; catalog no. 10566016; Thermo Fisher Scientific Inc., Waltham, MA, USA) containing 10% fetal bovine serum (FBS; catalog no. SH30396.03; Cytiva, Marlborough, MA, US), and 1% antibiotic–antimycotic mixed stock solution (catalog no. 161–23181; FUJIFILM Wako Pure Chemical Industries, Osaka, Japan). We cultured the fibroblasts at 37 ^°^C with 5% CO_2_.

### Staining of cytoskeleton and vimentin

Domestic and Iriomote cat fibroblasts were fixed with 4% paraformaldehyde in PBS) for 3 min. After three washes with PBS, cells were permeabilized with 0.5% Triton X-100 (35501–15; Nacalai Tesque) for 60 min. After washing with PBS, cells were incubated overnight with an anti-vimentin antibody (MA5–11883; Thermo Fisher Scientific). Cells were again washed with PBS and incubated with a secondary antibody (goat anti-mouse IgG (H + L) Cross-Adsorbed Secondary Antibody, Alexa Fluor 488; A-11001; Thermo Fisher Scientific). F-actin was simultaneously stained with Alexa Fluor 568 conjugated phalloidin (F-actin; dilution, A12380; Thermo Fisher, Waltham, MA, USA) for 45 min to stain the cellular cytoskeleton. We stained nuclei with 4’,6-diamidino- 2-phenylindole (DAPI) solution (340–07971; FUJIFILM Wako Pure Chemical Industries, Osaka, Japan). Stained images were observed using a BZ-9000 (Keyence, Tokyo, Japan).

### The time-course analysis of RIG-I, MDA5, TLR3, IL6, Mx, and OAS gene expressions after exposure of domestic and Iriomote wild cat-derived fibroblasts to poly(I:C)

Domestic and Iriomote cat-derived fibroblasts were incubated with 0 mg/mL, or 50 μg/mL poly(I:C) (cat no. 4287, TOCRIS a biotechne brand, Bristol, UK) in 5% CO_2_ at 37 ^°^C. After incubation, cells were washed with 1 × PBS, and total RNA was extracted. RNA collection points shows in Fig 3a. Total RNA were isolated from domestic- and Iriomote cat cells using NucleoSpin^®^ RNA (cat no. 740955.50, MACHEREY-NAGEL, Düren, Germany). Complementary DNA (cDNA) was synthesized with a PrimeScript reverse transcription (RT) reagent kit (Perfect Real Time, TaKaRa Bio, Kyoto, Japan). Quantitative polymerase chain reaction (qPCR) was performed according to the manufacturer’s protocol (KOD SYBR qPCR Mix; catalog no. QKD-201; TOYOBO; Osaka, Japan). In brief, qPCR reactions were performed in a 12.5 μL volume containing 2 × KOD SYBR qPCR Mix, 10 ng of cDNA, and 0.3 μM of each primer. Target gene expression levels were normalized to that of glyceraldehyde 3-phosphate dehydrogenase (*GAPDH*). The primers used in qPCR are described in S1 Table. This study analysis the gene expression of *RIG-I*, *MDA5*, *TLR3*, and downstream gene of *RIG-I* and *MDA5* (e.g., *IL6*, *Mx* and *OAS*).

**Fig 1:**
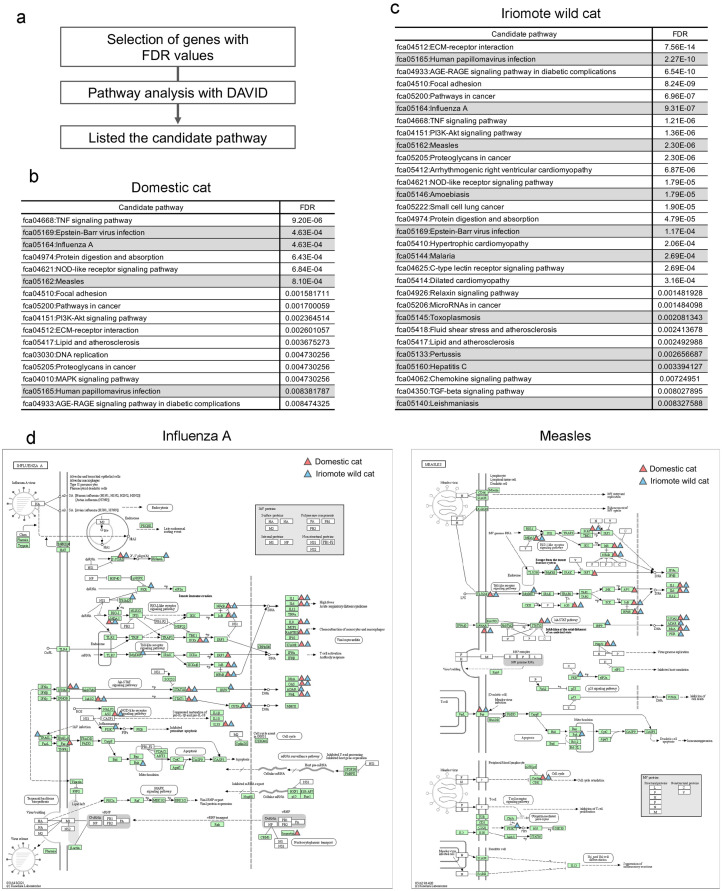
Candidate pathways exhibiting dramatic change with poly(I:C) stimulation in domestic cat and Iriomote wild cat derived fibroblasts. a: Candidate pathway analysis workflow. b, c: List of candidate pathways that exhibit dramatic change following poly(I:C) stimulation in domestic cat (b) and Iriomote wild cat (c) fibroblasts. Gray highlights indicate infectious disease-related pathways. We chose the candidate pathway with an FDR < 0.01. d: Visualization of extracted genes in the Influenza A (left side) and measles (right side) pathways. Red triangles indicate genes extracted from DEGs in domestic cat, Blue triangles indicate genes extracted from DEGs in Iriomote wild cat.

**Fig 2:**
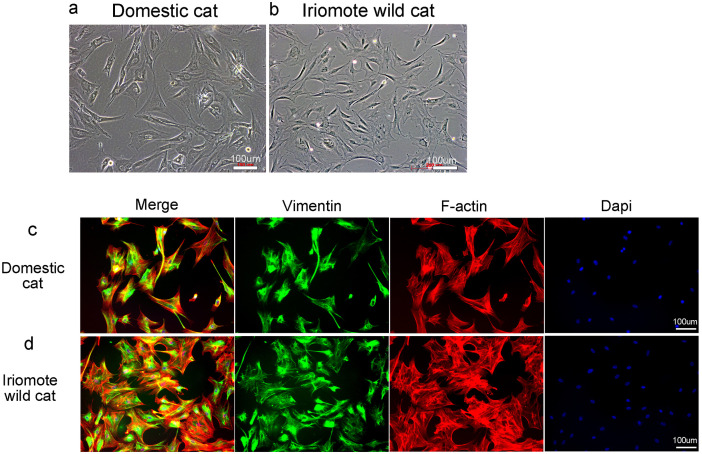
Cellular morphology and structure of domestic cat and Iriomote wild cat fibroblasts. a, b: Cellular morphology of domestic cat-skin derived fibroblasts (a) and iriomote wild cat-skin derived fibroblasts (b). The bars represent 100 μm. c, d: Structures of skin fibroblasts derived from domestic cats and Iriomote wild cats. The Left image is the merged image, the left middle image shows vimentin-staining, the right middle image shows F-actin-staining, and the right middle image displays DAPI-staining. The bars represent 100 μm.

### The analysis of three multiple individuals after exposure of domestic- and Iriomote wild cat-derived fibroblasts to poly(I:C)

Three individuals of domestic and Iriomote cat-derived fibroblasts were incubated with 0 mg/mL, 5 μg/mL, or 50 μg/mL poly(I:C) (cat no. 4287, TOCRIS a biotechne brand, Bristol, UK) in 5% CO_2_ at 37 ^°^C for 24 hours. After incubation, cells were washed with 1 × PBS, and total RNA was extracted. These flow shows in Fig 4a. Total RNA were isolated from domestic- and Iriomote cat cells using NucleoSpin^®^ RNA (cat no. 740955.50, MACHEREY-NAGEL, Düren, Germany). Complementary DNA (cDNA) was synthesized with a PrimeScript reverse transcription (RT) reagent kit (Perfect Real Time, TaKaRa Bio, Kyoto, Japan). Quantitative polymerase chain reaction (qPCR) was performed according to the manufacturer’s protocol (KOD SYBR qPCR Mix; catalog no. QKD-201; TOYOBO; Osaka, Japan). In brief, qPCR reactions were performed in a 12.5 μL volume containing 2 × KOD SYBR qPCR Mix, 10 ng of cDNA, and 0.3 μM of each primer. Target gene expression levels were normalized to that of glyceraldehyde 3-phosphate dehydrogenase (*GAPDH*). The primers used in qPCR are described in S1 Table. This study analysis the gene expression of *RIG-I*, *MDA5*, *TLR3*, and downstream gene of *RIG-I* and *MDA5* (e.g., *IL6*, *Mx* and *OAS*).

### RNA-seq analysis

Total RNA from domestic- and iriomote cat-derived fibroblasts was collected using NucleoSpin Tissue (740952.50; Macherey-Nagel). Fibroblast samples were analyzed in triplicate. To prepare our library, we used a TruSeq Stranded mRNA LT Sample Prep Kit (RS-122–2101; Illumina, San Diego, CA, USA). The cDNA samples were sequenced on an Illumina NovaSeq6000 sequencing machine, resulting in more than 40 M reads with 100 bp ends for each sample. We used CLC Genomic Workbench (CLC Bio, Aarhus, Denmark) to analyze the RNA-seq data. In the trim read step, sequence with quality scores below the CLC workbench threshold, 5’ ends, 3’ ends, and short sequences were removed. Trimmed sequence data were mapped onto the domestic cat reference genome using CLC Workbench. Gene expression data was obtained. PCA was performed using CLC Genomic Workbench with gene expression data. RNA-seq data from SRA (SRP090125 (PRJNA342639)) were used to compare cat cells. The RNA-seq data were submitted to the DNA DataBank of Japan under the accession numbers PRJDB17300 (BioProject), SAMD00729804-SAMD00729815 (BioSample).

To understand the differences in cellular characteristics between poly(I:C) exposed and non-exposed cells, we performed differentially expressed gene (DEG) analysis. We extracted approximately 1000 genes from DEGs in order of ascending FDR values between control (exposure to 0 μg/mL poly(I:C)) and exposed cells (50 μg/mL poly(I:C)) (Fig 6a). We first analyzed whether these 1000 genes were involved in the RIG-like receptor signaling pathway, as we have confirmed that poly:IC stimulates *RIG-I* and *MDA5* genes in domestic cat- and Iriomote wild cat-derived fibroblasts.

### Pathway analyses

To compare gene expression, we used the differential expression module in the RNA-Seq program of CLC Workbench. To identify differentially expressed genes, we used FDR. Based on FDR order, 1000 genes were selected. Candidate DEGs were processed using the DAVID pathway analysis tool.

We next searched for candidate pathways that changed dramatically after poly(I:C) exposure using the DAVID pathway analysis tool to assess the extracted genes (Fig 1a). Candidate pathways for domestic cats and Iriomote wild cats are shown in Fig 1b and c. Several infectious disease-related pathways, including influenza and measles were identified (Fig 1b,c). In particular, the Influenza A and Measles pathways showed significantly lower FDR values in both domestic cats and Iriomote wild cats compared to the other candidate pathways (Fig 1b,c). We here mapped the genes extracted from these pathways. Several extracted genes commonly mapped to Influenza A and Measles pathways (Fig 1d).

### Iriomote wild cat genome and amino acids

As the sequences of Iriomote wild cat *RIG-I*, *MDA5*, *TLR3* and downstream gene of *RIG-I* and *MDA5* (eg, *IL6*, *Mx*, and *OAS*) are not known, we attempted to obtain these sequences from a publicly available Iriomote wild cat draft genome publicly available in the DNA Databank of Japan (DDBJ). Because gene annotation has not yet been conducted (https://www.ncbi.nlm.nih.gov/datasets/genome/GCA_018403415.1/), we performed a BLAST search for *RIG-I*, *MDA5*, *TLR3*, *IL6*, *Mx*, and *OAS* using domestic cat *RIG-I*, *MDA5*, *TLR3*, *IL6*, *Mx*, and *OAS* sequences. We thereby obtained the Iriomote wild cat *RIG-I*, *MDA5*, *TLR3*, *IL6*, *Mx*, and *OAS* sequences. The sequences obtained were translated, and an amino acid sequence comparison with domestic cats was performed using Clustal Omega (https://www.ebi.ac.uk/Tools/msa/clustalo/).

### Statistical analyses

**Fig 3:**
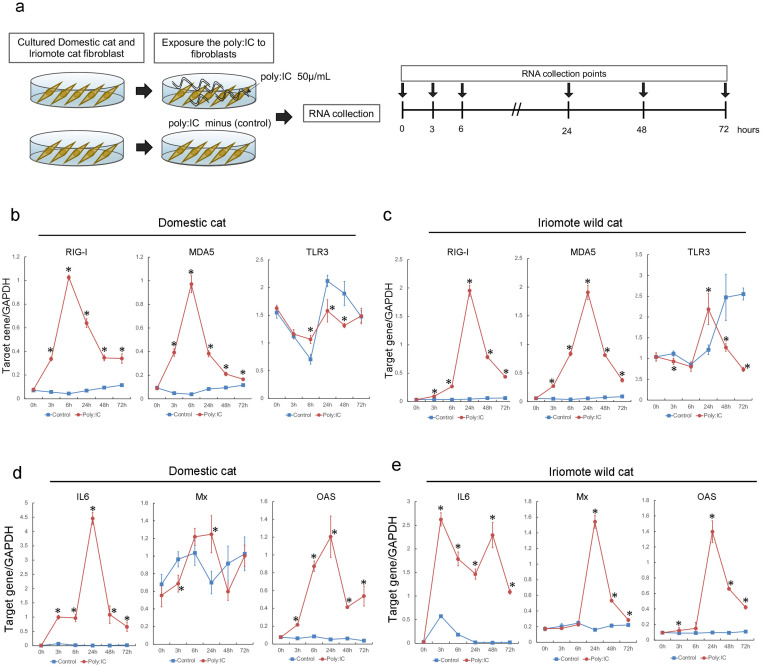
Time course analysis of gene expression after poly(I:C) exposure in domestic cat and Iriomote wild cat derived fibroblasts. a: Experimental flow of poly(I:C) exposure of fibroblasts derived from domestic cats and Iriomote wild cats. b, c: Time course analysis of *RIG-I*, *MDA5*, and *TLR3* gene expression after poly(I:C) exposure of domestic cat (b) and Iriomote wild cat (c) fibroblasts. The blue square indicates the control (exposure to 0 μg/mL poly(I:C)). The red circle indicates cells exposed to 50 μg/mL poly(I:C). Bars represent S.D. n = 6. *indicates p < 0.05. d, e: Time course analysis of *IL6*, *Mx*, and *OAS* gene expression after poly(I:C) exposure of domestic cat (d) and Iriomote wild cat (e) cells. Blue square shows control (0 μg/mL poly(I:C) exposure), Red circle shows 50 μg/mL poly:IC exposure. Bars indicate S.D. n = 6. *indicates p < 0.05.

**Fig 4:**
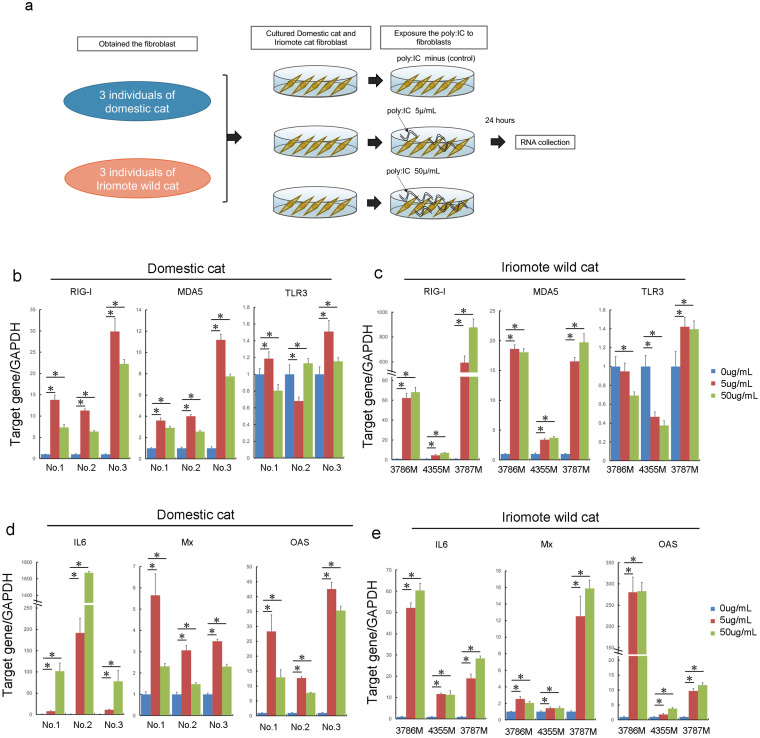
RIG-I, MDA5, TLR3, IL6, Mx, and OSA gene expression in each of three individual derived fibroblasts from domestic cat and Iriomote wild cat after exposure to poly(I:C). a: Experimental outline of domestic cat and Iriomote wild cat derived fibroblast exposure to poly(I:C). b, c: Expression of *RIG-I*, *MDA5*, and *TLR3* in domestic cats (b) and Iriomote wild cats (c) after exposure to poly(I:C). Blue bars show control (exposure to 0 μg/mL poly(I:C)), Red bars show 5 μg/mL poly:IC exposure, Green bars show 50 μg/mL poly(I:C) exposure. Error bars show standard deviation, n = 6. *shows p < 0.05. d, e: Expression of *IL6*, *Mx*, and *OAS* genes in domestic cat (d) and Iriomote wild cat (e) after exposure to poly(I:C). Blue bars indicate control (exposure to 0 μg/mL poly(I:C)), Red bars indicate 5 μg/mL poly(I:C) exposure, Green bars indicate 50 μg/mL poly(I:C) exposure. Error bars represent standard deviation, n = 6. *shows p < 0.05.

We first tested the normality of our dataset using the chi-square test for goodness of fit. A few data were not normally distributed (S2 and S3 Tables). Therefore, we employed a unified nonparametric analysis because it is not contingent on the normal distribution of the data. As shown in Fig 3b–e, the Mann–Whitney U test, which is a non-parametric analysis tool, was used to compare the two groups. In Fig 4b–e, we used the Steel test, which is a non-parametric analysis tool, to compare the control with the other two groups. Significant differences are indicated by *(p < 0.05). We used the statistical analysis software Statcel3 to perform these analyses (Statcel-the Useful Addin Forms on Excel-3rd ed., OMS Publishing, Higashi-Kurume, Tokyo, Japan).

## Results

### Cellular characteristics of domestic and Iriomote wild cat cultured fibroblasts

Although Iriomote wild cat-derived somatic cells are seldom available, skin-derived fibroblasts have been obtained from dead iriomote wild cats by our research group. Cultured fibroblasts have been used to analyze *RIG-I*, *MDA5*, *TLR3* and downstream gene of *RIG-I* and *MDA5* (e.g., *IL6*, *Mx*, and *OAS*) functions in several studies [8,11,14,15]. We compared the cellular characteristics of domestic- and Iriomote wild cat skin-derived cultured fibroblasts. We compared the cellular morphologies of domestic and wild Iriomote cat cells. Cellular morphologies differ slightly between domestic and Iriomote wild cats (Fig 2a,b). To examine cells in more depth, we stained vimentin (a marker of intermediate filaments) and F-actin (a marker of the actin cytoskeleton) in these cells. These two markers were similarly expressed in domestic- and Iriomote wild cat-cultured fibroblasts, therefore, these skin-derived fibroblasts have similar cellular structures in domestic and Iriomote wild cats (Fig 2c,d).

### The time-course analysis of *RIG-I*, *MDA5*, *TLR3*, *IL6*, *Mx*, and *OAS* gene expressions after exposure of domestic and Iriomote wild cat-derived fibroblasts to poly(I:C)

As shown in Fig 3a, we exposed domestic cat- and Iriomote wild-cat-derived fibroblasts to poly(I:C). *RIG-I* and *MDA5* expression consistently increased upon exposure to poly(I:C) in both domestic and Iriomote wild cat fibroblasts (Fig 3b,c). Therefore, poly(I:C) stimulates *RIG-I* and *MDA5* in domestic and Iriomote wild cat fibroblasts. Poly(I:C) stimulates *TLR3* in the fibroblasts of various animals [20–22]. In this study, although we observed significant changes at several time points, these changes with exposure to poly(I:C) were not consistent in domestic cat and Iriomote wild-cat fibroblasts (Fig 3b,c).

Downstream gene (*IL6*, *Mx*, and *OAS*) expression levels increased after exposure to poly(I:C) in both domestic and Iriomote wild cat fibroblasts (Fig 3d,e). Domestic cat *IL6*, domestic cat *OAS*, Iriomote wild cat *IL6*, Iriomote wild cat *Mx*, and Iriomote wild cat *OAS* dramatically increased after exposure to poly(I:C), similar to our observations with domestic and Iriomote wild cat *RIG-I* and *MDA5*.

### The analysis of three multiple individuals after exposure of domestic- and Iriomote wild cat-derived fibroblasts to poly(I:C)

We next exposed multiple individual derived domestic cat- and Iriomote wild-cat-derived fibroblasts to poly(I:C) (Fig 4a). *RIG-I* and *MDA5* expression consistently increased after poly(I:C) exposure in all domestic and Iriomote wild cats (Fig 4b,c). In contrast to *RIG-I* and *MDA5*, *TLR3* expression did not consistently increase upon exposure to poly(I:C) (Fig 4b,c). *IL6*, *Mx*, and *OAS*, which are downstream genes of *RIG-I* and *MDA5*, showed increased expression levels after poly(I:C) exposure in all individual domestic cat- and Iriomote wild cat-derived fibroblasts (Fig 4d,e).

### Comprehensive gene expression analysis after poly(I:C) exposure

**Fig 5:**
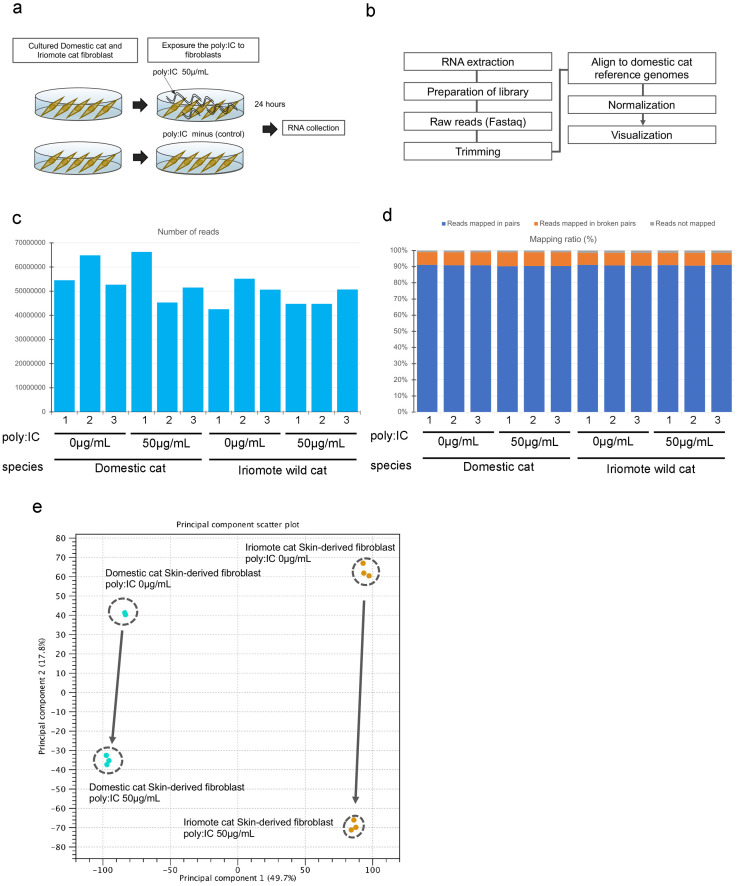
Comparison of comprehensive gene expression analyses of domestic cat and Iriomote wild cat derived fibroblasts after exposure to poly(I:C). a: Experimental flowchart of exposure of fibroblasts derived from domestic cat and Iriomote wild cat to poly(I:C). b: RNA-seq analysis workflow. c: Numbers of domestic cat- and Iriomote wild cat-derived fibroblasts. d: Mapping ratio for each sample. Blue bars: reads mapped in pairs; orange bars: reads mapped in broken pairs; grey bars: reads not mapped. e: PCA with profiling of Domestic cat fibroblasts treated with 0 μg/mL poly(I:C), Domestic cat fibroblasts treated with 50 μg/mL poly(I:C), Iriomote wild cat fibroblasts treated with 0 μg/mL poly(I:C), and Iriomote wild cat fibroblasts treated with 50 μg/mL poly(I:C).

We next analyzed the molecular responses of domestic cat- and Iriomote wild cat-derived fibroblasts after poly(I:C) exposure. As shown in Fig 5a, we exposed domestic cat- and Iriomote wild cat-derived fibroblasts to poly(I:C) and collected total RNA, with which we performed comprehensive gene expression analysis. A workflow chart is shown in Fig 5b. We obtained at least 40 M sequence reads, and consider this to be sufficient to describe the whole transcriptome (Fig 5c). In this study, we mapped both domestic cat and Iriomote wild cat read sequences to the domestic cat genome and obtained a mapping ratio for all cells of approximately 90% (Fig 5d). Processing of the data using Two-dimensional principal component analysis (PCA) revealed that the cellular characteristics of both domestic cat and Iriomote wild cat fibroblasts shifted in a similar direction after exposure to poly(I:C) (Fig 5e).

**Fig 6:**
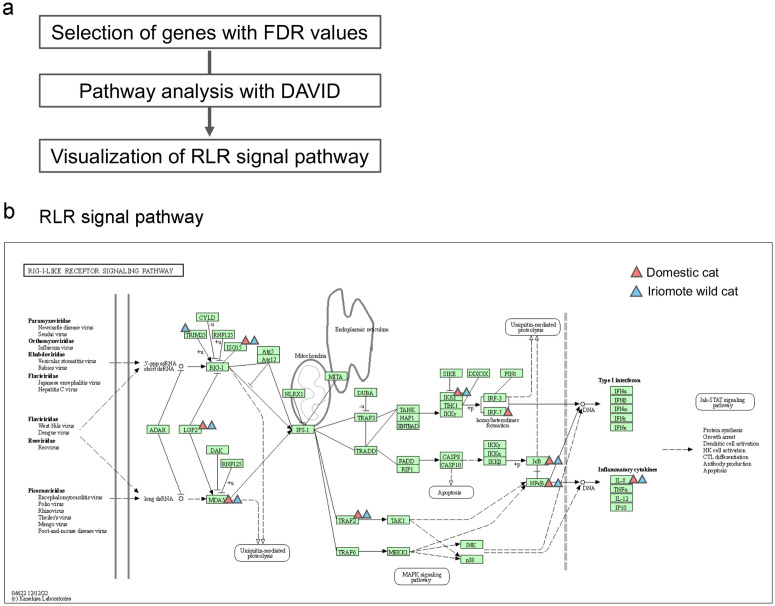
Mapping extracted genes to the RIG-I-like receptor signaling pathway. a: RNA-seq analysis workflow. b: Visualization of extracted genes in the RIG-I-like receptor signaling pathway. Red triangles indicate genes extracted from DEGs in domesticat, Blue triangles indicate genes extracted from DEGs in Iriomote wild cat.

We confirmed that a few of the extracted genes are involved in the RIG-like receptor signaling pathway (Fig 6b). Based on PCA results, a candidate pathway list, and mapping of the extracted genes in the Influenza A and Measles pathways, we concluded that similar responses occurred after poly:IC exposure in domestic cat- and Iriomote wild cat skin fibroblasts.

### Comparison of *RIG-I, MDA5, TLR3*, *IL6*, *Mx*, and *OAS* sequences in domestic cat and Iriomote wild cat

**Fig 7:**
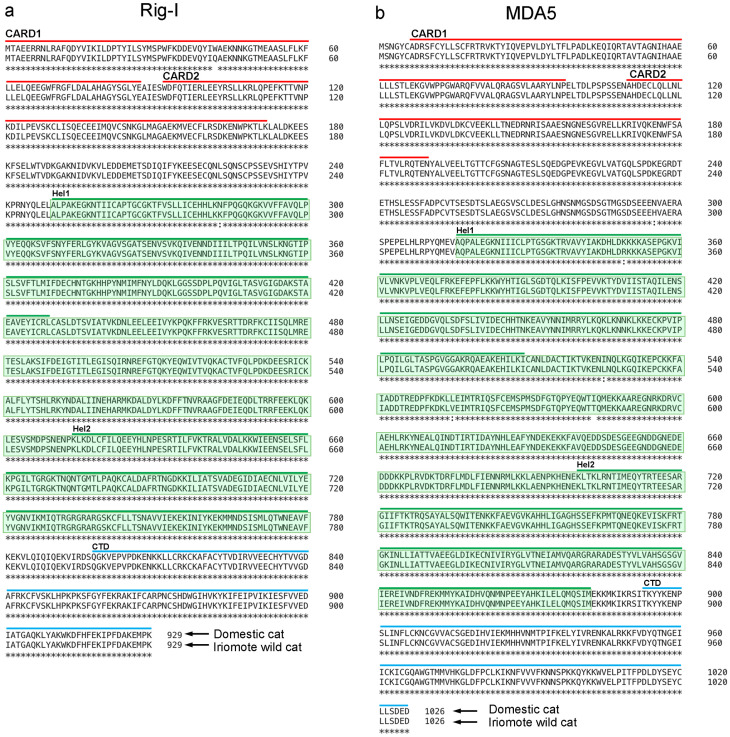
Comparison of amino acid homologies between Domestic cat and Iriomote wild cat RIG-I and MDA5 genes. a: Comparison of RIG-I amino acid homology between Domestic cats and Iriomote wild cats. b: Comparison of MDA5 amino acid homology between Domestic cats and Iriomote wild cats. Upper sequences are those of domestic cats, and lower sequences are those of Iriomote wild cats. Red lines show N-terminal tandem caspase activation and recruitment domains (CARDs). Green lines show the two central Rec A domains (Hel-1 and Hel-2) with DExH-box-type RNA helicase activity. Green highlighting indicates the RNA helicase region. The blue right lines represent the C-terminal domain (CTD).

**Fig 8:**
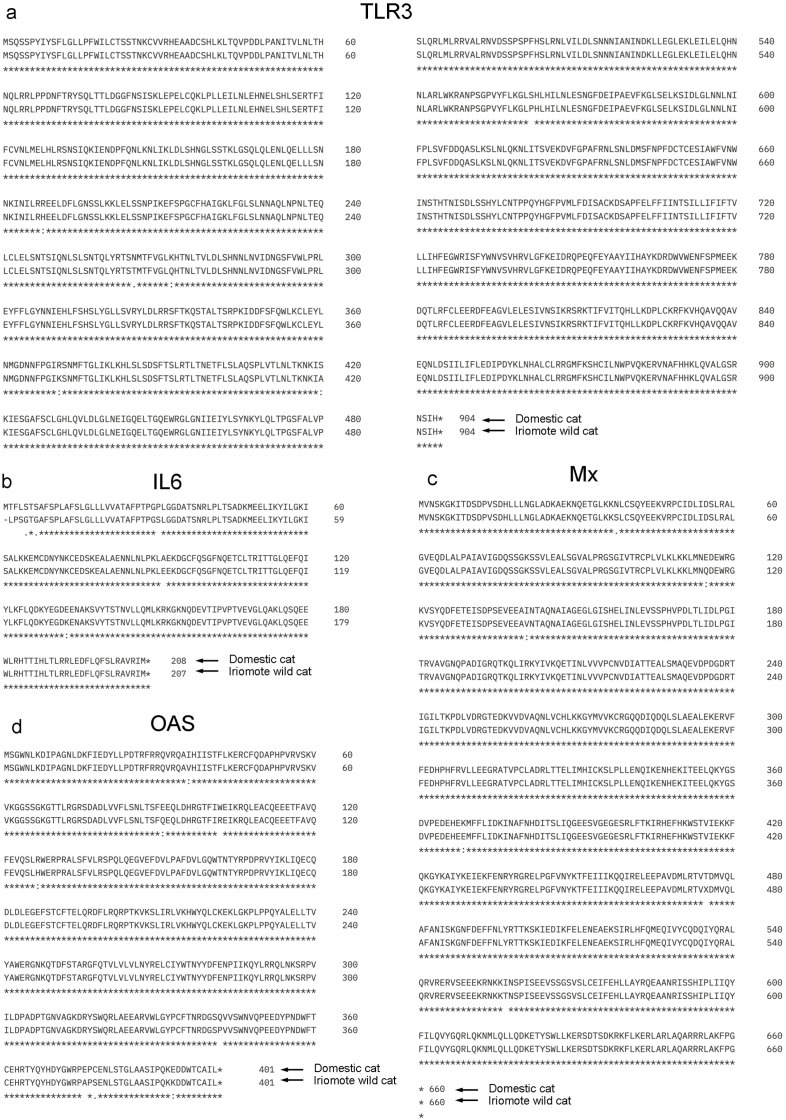
Comparison of amino acid homologies between Domestic cat and Iriomote wild cat TLR3, IL6, Mx, and OAS genes. a: Comparison of TLR3 amino acid homology between Domestic cats and Iriomote wild cats. b: Comparison of IL6 amino acid homology between Domestic cats and Iriomote wild cats. c: Comparison of Mx amino acid homology between Domestic cats and Iriomote wild cats. d: Comparison of OAS amino acid homology between Domestic cats and Iriomote wild cats.

We here compared the *RIG-I* and *MDA5* sequences in domestic cats and Iriomote wild cats. *RIG-I* and *MDA5* sequences are highly conserved between domestic cat and Iriomote wild cat (Fig 7a,b). Specifically, iriomote wild cat *MDA5* vs. domestic cat *MDA5*; 927/929, iriomote wild cat *RIG-I* vs. domestic cat *RIG-I*; 1021/1026. These results support our conclusion that similar responses occur in domestic cat and Iriomote wild-cat fibroblasts after poly(I:C) exposure, since poly(I:C) stimulates *RIG-I* and *MDA5*. We next compared the *TLR3* sequences in domestic cats and Iriomote wild cats. *TLR3* sequences are highly conserved between domestic cat and Iriomote wild cat (Fig 8a). Iriommote wild cat *TLR3* vs. domestic cat *TLR3*; 898/904. Furthermore, we also compared the *IL6*, *Mx*, and *OAS* sequences in domestic cats and Iriomote wild cats (Fig 8b,c,d). Iriomote wild cat *IL6* vs. domestic cat *IL6*; 199/208, Iriomote wild cat *Mx* vs. domestic cat *Mx*; 655/660, Iriomote wild cat *OAS* vs. domestic cat *OAS*; 393/401.

## Discussion

In this study, we exposed domestic and Iriomote wild cat-derived fibroblasts to poly(I:C). *RIG-I* and *MDA5* expression increased in both domestic cat and Iriomote wild cat fibroblasts after exposure. We thus concluded that poly(I:C) can stimulate *RIG-I* and *MDA5* genes in both domestic cat and Iriomote wild cat fibroblasts. In contrast to *RIG-I* and *MDA5*, *TLR3* gene expression did not change dramatically after exposure to poly(I:C). We thus considered that poly(I:C) mainly stimulates *RIG-I* and *MDA5* among these three genes in this study.

Poly(I:C) is an analog of double-stranded RNA. Therefore, similar to viral RNA, poly I: C is recognized by the C-terminal domain (CTD) of *RIG-I* and *MDA5* [23–25]. The sequences of the CTD regions of *RIG-I* and *MDA5* are perfectly conserved between the domestic cat and the Iriomote wild cat. Therefore, Iriomote wild cat RIG-I and MDA5 recognize poly(I:C) similarly to domestic cat RIG-I and MDA5. We here confirmed that poly(I:C) stimulates *RIG-I* and *MDA5* genes in domestic cat and Iriomote wild cat fibroblasts. We thus consider *RIG-I* and *MDA5* to be activated through recognition of poly(I:C) in domestic cat and Iriomote wild cat fibroblasts.

We analyzed in detail the molecular response of domestic cat and Iriomote wild cat fibroblasts after poly(I:C) exposure. In this study, we determined that the molecular response after poly(I:C) exposure is mainly *RIG-I* and *MDA5*-derived signals, since *TLR3* gene expression levels did not dramatically change after exposure to poly(I:C) compared to *RIG-I* and *MDA5*. According to PCA results, although there was no perfect match, the cellular characteristics of both domestic cat and Iriomote wild cat fibroblasts moved in a similar direction upon exposure to poly(I:C). We thus concluded that the molecular responses to poly(I:C) exposure will be similar between domestic and Iriomote wild cats.

Our previous study shows that poly(I:C) could stimulate the *RIG-I*, *MDA5*, and *TLR3* of chicken and Okinawa rai fibroblast in the transfection reagent minus medium [14]. In contrast to the previous study, *TLR3* expression is not dramatically changed in either domestic cat or Iriomote wild cat cells in this study (Fig 4). There are unclear whether the differences are due to a species difference or a cellular difference. Therefore, we tried to analyze the *TLR3* gene expression in domestic cat ovarian-derived fibroblasts after poly(I:C) exposure without the transfection reagent. We confirmed that the increase in *TLR3* gene expression in domestic cat ovarian-derived fibroblasts occurred without the use of a transfection reagent (S1 Fig). Therefore, the reason why *TLR3* expression is not dramatically changed in either domestic cat or Iriomote wild cat cells in this study would be cellular differences.

We searched for candidate pathways that changed dramatically after exposure to poly(I:C) in both domestic cat and Iriomote wild-cat fibroblasts, and identified many infectious disease-related pathways, including influenza A. *RIG-I* and *MDA5*-derived signals are critical for host defense via the innate immune response [26–28]. The innate immune response and infectious diseases are closely related; therefore, many infectious disease-related pathways have been listed as candidate pathways for the response to poly(I:C) exposure.

*RIG-I* and *MDA5* play important roles in host defense as viral RNA sensors. *RIG-I* and *MDA5*-derived signals have a critical effect on the exacerbation of infectious diseases with the virus in various animals. Studies of the Iriomote wild cat are rare regarding cellular response. Our results provide useful information for predicting viral responses in Iriomote wild cats.

## Conclusion

We concluded that *RIG-I* and *MDA5*-mediated molecular responses to poly(I:C) exposure are similar in domestic and Iriomote wild cats’ fibroblasts. To the best of our knowledge, this is the first study to compare the *RIG-I* and *MDA5* response to poly(I:C) between Iriomote wild cats and domestic cats. *RIG-I* and *MDA5* are important sensors for recognizing viruses and triggering host defense responses, including activation of the innate immune response. Our results provide useful information for Iriomote wild cat conservation efforts, as studies of cellular response are rare.

## Supporting information

S1 Fig RIG-I, MDA5 and TLR3 genes expression. Expression of RIG-I, MDA5, and TLR3 in domestic cats ovary derived fibroblast after exposure to poly(I:C). We used one individual-derived ovary-derived fibroblast in this study. Blue bars show control (exposure to 0 μg/mL poly(I:C)), Red bars show 5 μg/mL poly:IC exposure, Green bars show 50 μg/mL poly(I:C) exposure. Error bars show standard deviation, n = 6.
(JPG)

S1 Table Primer sequence information of real-time PCR. Detailed information is shown in the table.
(PDF)

S2 Table Chi-square test in Fig 3. Detailed information is shown in the table.
(PDF)

S3 Table Chi-square test in Fig 4. Detailed information is shown in the table.
(PDF)
