# Supplementary figures and images for: Comparison of cellular responses of cultured fibroblasts from Iriomote wild cats and domestic cats exposure to polyinosinic:polycytidylic acid
Source: PLoS One. 2025 Sep 25;20(9):e0332954. doi: 10.1371/journal.pone.0332954 (PMC12463245; doi:10.1371/journal.pone.0332954)

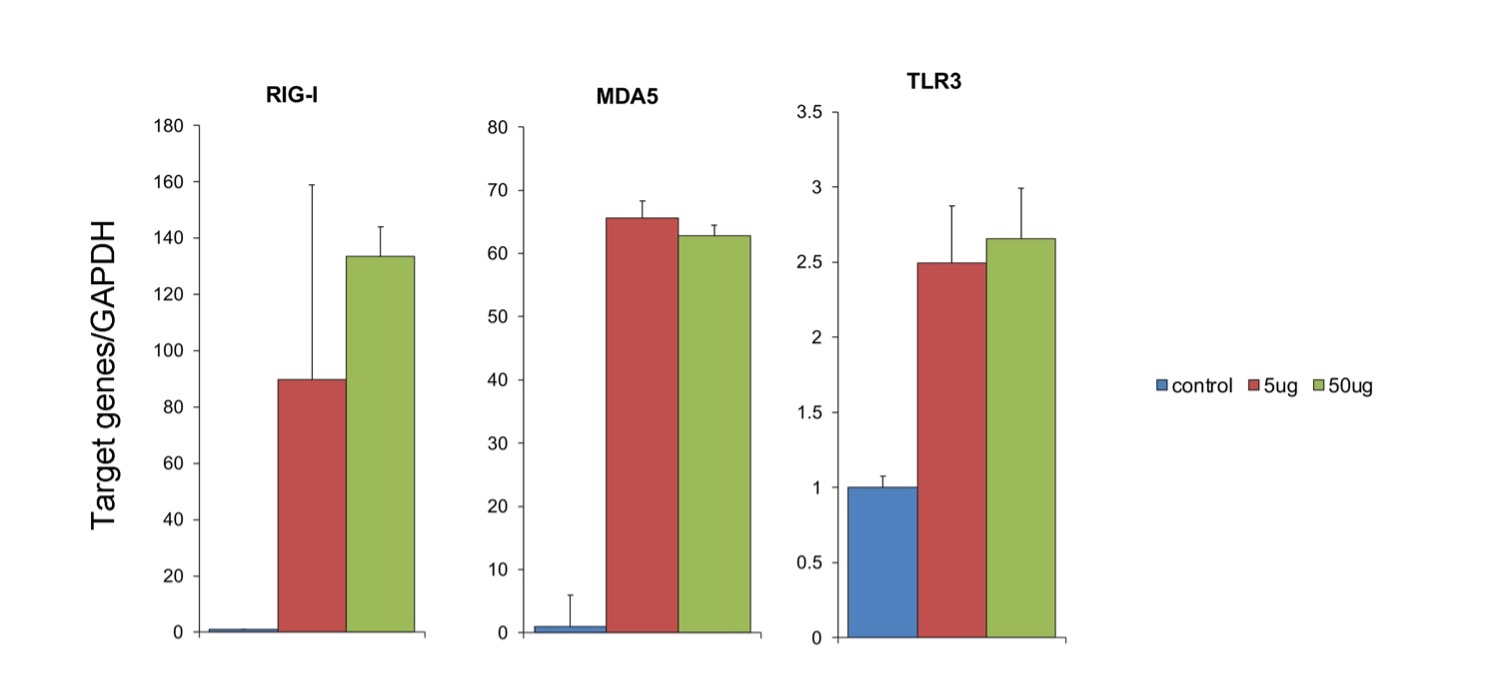

Supplement: S1 Fig — Expression of RIG-I, MDA5, and TLR3 in domestic cats ovary derived fibroblast after exposure to poly(I:C). We used one individual-derived ovary-derived fibroblast in this study. Blue bars show control (exposure to 0 μg/mL poly(I:C)), Red bars show 5 μg/mL poly:IC exposure, Green bars show 50 μg/mL poly(I:C) exposure. Error bars show standard deviation, n = 6. (JPG) [file pone.0332954.s001.jpg]
