# Supplementary material for: Comparison of cellular responses of cultured fibroblasts from Iriomote wild cats and domestic cats exposure to polyinosinic:polycytidylic acid
Source: PLoS One. 2025 Sep 25;20(9):e0332954. doi: 10.1371/journal.pone.0332954 (PMC12463245; doi:10.1371/journal.pone.0332954)
Supplement: S1 Table — Detailed information is shown in the table. (PDF) [file pone.0332954.s002.pdf]

Gene name	F/R	Sequence	bp
RIG-I	Foward	AGCCAAGAAATTACCAACTAGAGC	84
	Reverse	TTCCACAACCAGTAGGAGCAC	
MDA5	Foward	ACTGCATCTCAGGCCCTACCAA	104
	Reverse	CGGCCACTCTGGTTTTCCCACT	
TLR3	Foward	GAACAGAACCTCTAAATATTGCCTC	133
	Reverse	GTTGATGACCAATATGGCTTCCTC	
IL6	Foward	AGACCTGCCTGACAAGAATCACT	99
	Reverse	ACTTGGCATTTTCCTTATCACCCTC	
Mx1	Foward	ACATCGGACGCCAGACCAAGC	146
	Reverse	CCTGTCTCCATCGGGGTCCAC	
OAS	Foward	CCTCGTGGTCTTTCTGAGTAACCT	141
	Reverse	AGACTCTGTACTTCAAACTGCACT	
GAPDH	Foward	GCTTTTAACTCTGGCAAAGTGGAC	146
	Reverse	TTGATGACAAGTTTCCCGTTCTCAG	
