# Supplementary material for: Comparison of cellular responses of cultured fibroblasts from Iriomote wild cats and domestic cats exposure to polyinosinic:polycytidylic acid
Source: PLoS One. 2025 Sep 25;20(9):e0332954. doi: 10.1371/journal.pone.0332954 (PMC12463245; doi:10.1371/journal.pone.0332954)
Supplement: S2 Table — Detailed information is shown in the table. (PDF) [file pone.0332954.s003.pdf]

Fig number	Species	Gene name			normal distribution
Fig.2b	Domestic cat	RIG-I	contol	0h	Yes
				3h	Yes
				6h	Yes
				24h	Yes
				48h	Yes
				72h	Yes
			poly:IC	0h	Yes
				3h	Yes
				6h	Yes
				24h	Yes
				48h	Yes
				72h	Yes
		MDA5	contol	0h	Yes
				3h	Yes
				6h	Yes
				24h	Yes
				48h	Yes
				72h	Yes
			poly:IC	0h	Yes
				3h	Yes
				6h	Yes
				24h	Yes
				48h	Yes
				72h	Yes
		TLR3	contol	0h	Yes
				3h	Yes
				6h	Yes
				24h	Yes
				48h	Yes
				72h	Yes
			poly:IC	0h	Yes
				3h	Yes
				6h	Yes
				24h	Yes
				48h	Yes
				72h	Yes

Fig number	Species	Gene name			normal distribution
Fig.2c	Iriomote wild cat	RIG-I	contol	0h	Yes
				3h	Yes
				6h	Yes
				24h	Yes
				48h	Yes
				72h	Yes
			poly:IC	0h	Yes
				3h	Yes
				6h	Yes
				24h	Yes
				48h	Yes
				72h	Yes
		MDA5	contol	0h	Yes
				3h	Yes
				6h	Yes
				24h	Yes
				48h	Yes
				72h	Yes
			poly:IC	0h	Yes
				3h	Yes
				6h	Yes
				24h	Yes
				48h	Yes
				72h	Yes
		TLR3	contol	0h	Yes
				3h	Yes
				6h	Yes
				24h	Yes
				48h	Yes
				72h	Yes
			poly:IC	0h	Yes
				3h	Yes
				6h	Yes
				24h	Yes
				48h	Yes
				72h	No

Fig number	Species	Gene name			normal distribution
Fig.2d	Domestic cat	IL6	contol	0h	Yes
				3h	Yes
				6h	Yes
				24h	Yes
				48h	Yes
				72h	Yes
			poly:IC	0h	Yes
				3h	Yes
				6h	Yes
				24h	Yes
				48h	Yes
				72h	Yes
		Mx	contol	0h	Yes
				3h	Yes
				6h	Yes
				24h	Yes
				48h	Yes
				72h	Yes
			poly:IC	0h	Yes
				3h	Yes
				6h	Yes
				24h	Yes
				48h	Yes
				72h	Yes
		OAS	contol	0h	Yes
				3h	Yes
				6h	Yes
				24h	Yes
				48h	Yes
				72h	Yes
			poly:IC	0h	Yes
				3h	Yes
				6h	Yes
				24h	Yes
				48h	Yes
				72h	Yes

Fig number	Species	Gene name			normal distribution
Fig.2c	Iriomote wild cat	IL6	contol	0h	Yes
				3h	Yes
				6h	Yes
				24h	Yes
				48h	Yes
				72h	Yes
			poly:IC	0h	Yes
				3h	Yes
				6h	Yes
				24h	Yes
				48h	Yes
				72h	Yes
		Mx	contol	0h	Yes
				3h	Yes
				6h	Yes
				24h	Yes
				48h	Yes
				72h	Yes
			poly:IC	0h	Yes
				3h	Yes
				6h	Yes
				24h	Yes
				48h	Yes
				72h	Yes
		OAS	contol	0h	Yes
				3h	Yes
				6h	Yes
				24h	Yes
				48h	Yes
				72h	Yes
			poly:IC	0h	Yes
				3h	No
				6h	Yes
				24h	Yes
				48h	Yes
				72h	Yes
