# Supplementary material for: Comparison of cellular responses of cultured fibroblasts from Iriomote wild cats and domestic cats exposure to polyinosinic:polycytidylic acid
Source: PLoS One. 2025 Sep 25;20(9):e0332954. doi: 10.1371/journal.pone.0332954 (PMC12463245; doi:10.1371/journal.pone.0332954)
Supplement: S3 Table — Detailed information is shown in the table. (PDF) [file pone.0332954.s004.pdf]

Fig number	Species	Gene name	Gene name	Dose	normal distribution
				0μg/mL	Yes
			No.1	5μg/mL	Yes
				50μg/mL	Yes
				0μg/mL	Yes
		RIG-I	No.2	5μg/mL	Yes
				50μg/mL	Yes
				0μg/mL	No
			No.3	5μg/mL	Yes
				50μg/mL	Yes
				0μg/mL	Yes
			No.1	5μg/mL	Yes
				50μg/mL	Yes
				0μg/mL	Yes
Fig.3b	Domestic cat	MDA5	No.2	5μg/mL	Yes
				50μg/mL	Yes
				0μg/mL	Yes
			No.3	5μg/mL	Yes
				50μg/mL	Yes
				0μg/mL	Yes
			No.1	5μg/mL	Yes
				50μg/mL	Yes
				0μg/mL	Yes
		TLR3	No.2	5μg/mL	Yes
				50μg/mL	Yes
				0μg/mL	Yes
			No.3	5μg/mL	Yes
				50μg/mL	Yes
				0μg/mL	Yes
			3786M	5μg/mL	Yes
				50μg/mL	No
				0μg/mL	Yes
		RIG-I	4355M	5μg/mL	Yes
				50μg/mL	No
				0μg/mL	Yes
			3787M	5μg/mL	Yes
				50μg/mL	Yes
				0μg/mL	Yes
			3786M	5μg/mL	Yes
				50μg/mL	Yes
				0μg/mL	Yes
Fig.3c	Iriomote wild cat	MDA5	4355M	5μg/mL	Yes
				50μg/mL	Yes
				0μg/mL	Yes
			3787M	5μg/mL	Yes
				50μg/mL	Yes
				0μg/mL	Yes
			3786M	5μg/mL	Yes
				50μg/mL	Yes
				0μg/mL	Yes
		TLR3	4355M	5μg/mL	Yes
				50μg/mL	Yes
				0μg/mL	Yes
			3787M	5μg/mL	Yes
				50μg/mL	Yes

Fig number	Species	Gene name	Gene name	Dose	normal distribution
				0μg/mL	Yes
			No.1	5μg/mL	Yes
				50μg/mL	Yes
				0μg/mL	Yes
		IL6	No.2	5μg/mL	Yes
				50μg/mL	Yes
				0μg/mL	Yes
			No.3	5μg/mL	Yes
				50μg/mL	Yes
				0μg/mL	No
			No.1	5μg/mL	Yes
				50μg/mL	Yes
				0μg/mL	Yes
Fig.3d	Domestic cat	Mx	No.2	5μg/mL	Yes
				50μg/mL	Yes
				0μg/mL	Yes
			No.3	5μg/mL	Yes
				50μg/mL	Yes
				0μg/mL	Yes
			No.1	5μg/mL	Yes
				50μg/mL	Yes
				0μg/mL	Yes
		OAS	No.2	5μg/mL	Yes
				50μg/mL	Yes
				0μg/mL	Yes
			No.3	5μg/mL	Yes
				50μg/mL	Yes
				0μg/mL	Yes
			3786M	5μg/mL	Yes
				50μg/mL	Yes
				0μg/mL	Yes
		IL6	4355M	5μg/mL	Yes
				50μg/mL	Yes
				0μg/mL	Yes
			3787M	5μg/mL	Yes
				50μg/mL	Yes
				0μg/mL	Yes
			3786M	5μg/mL	Yes
				50μg/mL	Yes
				0μg/mL	Yes
Fig.3e	Iriomote wild cat	Mx	4355M	5μg/mL	Yes
				50μg/mL	Yes
				0μg/mL	Yes
			3787M	5μg/mL	Yes
				50μg/mL	Yes
				0μg/mL	Yes
			3786M	5μg/mL	Yes
				50μg/mL	Yes
				0μg/mL	Yes
		OAS	4355M	5μg/mL	No
				50μg/mL	Yes
				0μg/mL	Yes
			3787M	5μg/mL	Yes
				50μg/mL	Yes
